# Does the gut microbiome environment influence response to systemic breast cancer treatment?

**DOI:** 10.37349/etat.2021.00051

**Published:** 2021-08-30

**Authors:** Eilidh Bruce, Stanislau Makaranka, Gordon Urquhart, Beatrix Elsberger

**Affiliations:** 1 Department of Breast Surgery, Aberdeen Royal Infirmary, NHS Grampian, AB25 2ZN Aberdeen, Scotland, UK; 2 University of Aberdeen, AB25 2ZN Aberdeen, Scotland, UK; 3 Department of Oncology, Aberdeen Royal Infirmary, NHS Grampian, AB25 2ZN Aberdeen, Scotland, UK; University of Southampton, UK

**Keywords:** Breast cancer, gut microbiome, gut microbiota, dysbiosis, estrobolome

## Abstract

The gut microbiome is a novel player in the pathogenesis and treatment of breast cancer. The term “microbiome” is used to describe the diverse community of micro-organisms existing within the gastrointestinal tract. The gut microbiome plays an important role in oestrogen metabolism through its ability to deconjugate oestrogens within the gut resulting in their reabsorption. Therefore, it is not unsurprising that “dysbiosis”, the disruption of normal gut microbiota composition, is now thought to play a role in the development of the disease, as women with breast cancer have been shown to have altered gut microbiota and this has been correlated with tumour characteristics. There is emerging evidence to suggest that the gut microbiota may also impact on breast cancer treatment, by mediating both drug efficacy and toxicity. The present review will discuss the influence of the gut microbiota on systemic treatments for breast cancer, including chemotherapy, anti-human epidermal growth factor receptor 2 (HER2) therapy, endocrine therapy and immunotherapy as well as other targeted treatments.

## Introduction

Breast cancer remains the most common cancer in women in the UK, accounting for 15% of all new cancer cases, with an annual incidence of 55,200 [1]. It accounts for the 4th most common cause of cancer mortality in the UK, with 11,400 breast cancer deaths every year [1]. However, due to advances in both diagnosis and treatment, mortality rates over the last decade have steadily declined by 21% in the UK [1].

Well established risk factors for breast cancer, such as nulliparity, use of hormonal therapies and positive family history, are only present in around 50% of those diagnosed [2]. However, no specific risk factors for the development of breast cancer are identified in the remaining 50% of patients. Yet a novel player in the pathogenesis and treatment of breast cancer is being recognised—the gut microbiome. The “gut microbiota” within the human gastrointestinal (GI) tract is a diverse community of trillions of microorganisms, amounting to over 1,000 different species of viruses, bacteria, fungi, and archaea [3]. The host and microbiota engage in a symbiotic relationship to maintain normobiosis; however, a state of dysbiosis may promote disease pathogenesis [4, 5].

Literature suggests that a state of dysbiosis, that is the disruption of the gut microbiota, may play a role in the pathogenesis of breast cancer. Postmenopausal women with breast cancer have been shown to possess a gut microbiota of altered composition and reduced diversity when compared to healthy controls [6]. Other studies have also implicated differences in microbiota of women with breast cancer compared to controls. Luu et al. [7], for example, showed that intestinal microbiota differs according to tumour characteristics as well as body mass index (BMI). Specifically, patients with histological Grade 3 breast tumours had increased numbers of *Blautia* species within the gut when compared to patients with Grade 1 cancers [7].

When considering factors that influence the gut microbiota, method of delivery at birth, infant feeding experience, genetics, infections, medications, diet and smoking are at the forefront [8]. Indeed, diet, for example, has been shown to rapidly and reproducibly alter the composition of human gut microbiota [9]. The link between diet and obesity has also been demonstrated to be mediated by the gut microbiota, rather than exclusively driven by high calorie diet [8]. This highlights the importance of diet and lifestyle factors in the make-up of the gut microbiota. These can be easily overlooked, but have potential to be used as a treatment target for patients with breast cancer. Furthermore, it is currently unknown whether cancer pathology leads to alteration in the gut microbiota or whether dysbiosis in itself is the carcinogenic factor.

There is emerging evidence to suggest that dysbiosis may not only play a role in the pathogenesis of breast cancer, but may also influence response to systemic treatment of the disease. Microbiota interacts with breast cancer treatments in a plethora of ways, which can influence the degree of efficacy of chemotherapeutic drugs and immune therapies as well as the side effects from such therapies [10]. We present a review of the current evidence for the role of the gut microbiome in influencing systemic breast cancer therapy, specifically with reference to the bacterial component of the microbiota.

## Chemotherapy

The gut microbiota is thought to modulate chemotherapeutic agents through a number of key mechanisms, namely translocation, immunomodulation, metabolism, enzymatic degradation and reduced diversity and ecological variation (known as the TIMER framework) [11]. The impact that these processes have on host response to chemotherapeutic drugs are grouped into three categories: facilitation of drug efficacy, compromise of anti-cancer effects and/or mediation of toxicity [11].

Translocation, the process through which bacteria pass across the gut barrier into the systemic circulation, allows species of the gut microbiota to possess the potential to contribute to chemotherapy’s morbidity and also improve its’ efficacy [12]. Literature in this field relates to the study of haematological malignancy rather than malignant disease of the breast. However, in mice models, both doxorubicin and cyclophosphamide have been shown to induce the translocation of selected Gram-positive bacteria into secondary lymphoid organs [13]. Once translocation has occurred, a specific subset of T helper 17 cells and memory T helper 1 cells are generated, which contribute to the aforementioned chemotherapy agents’ efficacy [13]. Conversely, evidence exists to support sterile mice or mice which have been treated with antibiotics to reduce the diversity of the microbiota had tumours which were resistant to the effects of chemotherapy [13].

With regard to immunomodulation, Iida et al. [14] showed that commensal bacteria also impact response to chemotherapy by modulating the microenvironment of subcutaneous tumours. In sterile mice or those treated with antibiotics, tumour-infiltrating myeloid-derived cells had a poor response to platinum chemotherapy resulting from poor cytokine production [14]. However, those mice treated with CpG-oligonucleotides (CpG-ODN) displayed tumour necrosis with reduced production of reactive oxygen species and less cytotoxicity [14].

The gut microbiota not only impacts the therapeutic efficacy of chemotherapy, but also is thought to have an impact on its toxic effects. Mucositis, often manifesting itself as diarrhoea, is a common adverse effect of chemotherapy, which causes morbidity and mortality [15]. As a result of systemic chemotherapy, the gut microbiota demonstrates reduced diversity and ecological variation patterns [11]. Namely a reduction in gut villous length and a reduction in diversity of the microbiota have been shown [16]. Patients receiving chemotherapy for haematological malignancy is thought to have reduced levels of *Bifidobacterium*, *Clostridium* cluster XIV5, *Faecalibacterium prausnitzii* within the GI tract, and increased levels of *Enterobacteriaceae* and *Bacteroides* [16]. The increased presence of these species may contribute to the development of mucositis, weight loss and bacteraemia [16], through metabolism and enzymatic degradation of the chemotherapeutic agents. However, the impact of gut microbiota on the toxic effects specific to breast cancer chemotherapeutic agents is yet to be examined. Randomised controlled trials have been performed to investigate the efficacy of probiotics in preventing cancer therapy-induced mucositis with 3 out of 6 trials showing a significant reduction in the incidence of diarrhea [16].

In terms of emergent interventions currently available, most are aimed at reducing toxicity from chemotherapy rather than improving efficacy. A study in mice demonstrated that a diet high in protein, *L*-leucine, fish oil and specific oligosaccharides can reduce the incidence and severity of *Pseudomonas* translocation from cyclophosphamide-induced immunosuppression, for example [17]. 5-fluorouracil (5-FU) has been shown to have an enhanced effect in the presence of ginseng in colorectal cancer treatment; however, it is not known whether the same can be said for breast cancer [18]. Polysaccharides from the ink of squid have been shown to enrich the levels of bifidobacteria and reduce levels of *Bacteroidetes*, again being a potential treatment option to reduce adverse effects of chemotherapy [19].

Probiotics, prebiotics and synbiotics are another treatment strategy to reduce the risk of mucositis during chemotherapy [20]. Meanwhile, prebiotics, namely oligofructose and inulin, have been shown to both inhibit malignant tumour growth and potentiate the effects of six different chemotherapeutic agents in animal models [21].

### Anti-huamn epidermal growth factors receptor 2 therapy

There is strong evidence that the composition of the gut microbiota can also potentiate the effects or reduce the efficacy of anti-huamn epidermal growth factors receptor 2 (HER2) therapies in breast cancer. Di Modica et al. [22] studied the relationship between commensal bacteria composition and clinical efficacy of trastuzumab in HER2 positive patients, finding that administration of antibiotics impaired the efficacy of anti-HER2 therapy. The effect was achieved through reducing the CD4^+^ T cells’ and natural killer (NK) cells’ recruitment due to reduced bacterial biodiversity in the gut; mice transplanted with faeces of antibiotic-treated mice also did not benefit from anti-HER2 treatment, thus supporting the initial findings [22]. Mice who achieved a complete pathological tumour response to anti-HER2 therapy had a significantly higher microbial diversity in the gut and non-responders had reduced Clostridiales and increased *Bacteroidales* species [22]. These represent potential treatment strategies to enhance the therapeutic efficacy of trastuzumab (through manipulation of intestinal flora).

Another study in patients found two microbiota clusters, namely Clostridiales and *Bacteroides,* which discriminated patients according to whether they had a complete response to anti-HER2 therapy [23]. Responders were found to have a higher abundance of Clostridiales and lower abundance of *Bacteroides*, supporting the evidence provided by the study discussed above [22, 23]. Furthermore, in the same study, intestinal flora-depleted mice were transplanted with faecal material from responders and non-responders patients. The mice’s response to trastuzumab was identical to that of patients from which stool was obtained [23]. This suggests a causal relationship between gut microbiota and response to anti-HER2 therapies, independent of tumour molecular characteristics. Manipulating the gut microbiota could thus increase the efficacy of trastuzumab, perhaps through transplantation of faecal material as done in the study discussed above. The gut microbiota could also be used as a marker of treatment response, through measurement of the different species of bacteria within it.

### Endocrine therapy

It is well known that the gut microbiota plays a role in oestrogen metabolism [24]. The collective numbers of bacterial genes in the GI tract that are capable of metabolizing oestrogens have been named the “Estrobolome” [25]. This “aggregate of enteric bacterial genes” has been considered as a potential factor which can modulate risk of oestrogen receptor positive breast cancer.

**Figure 1.**
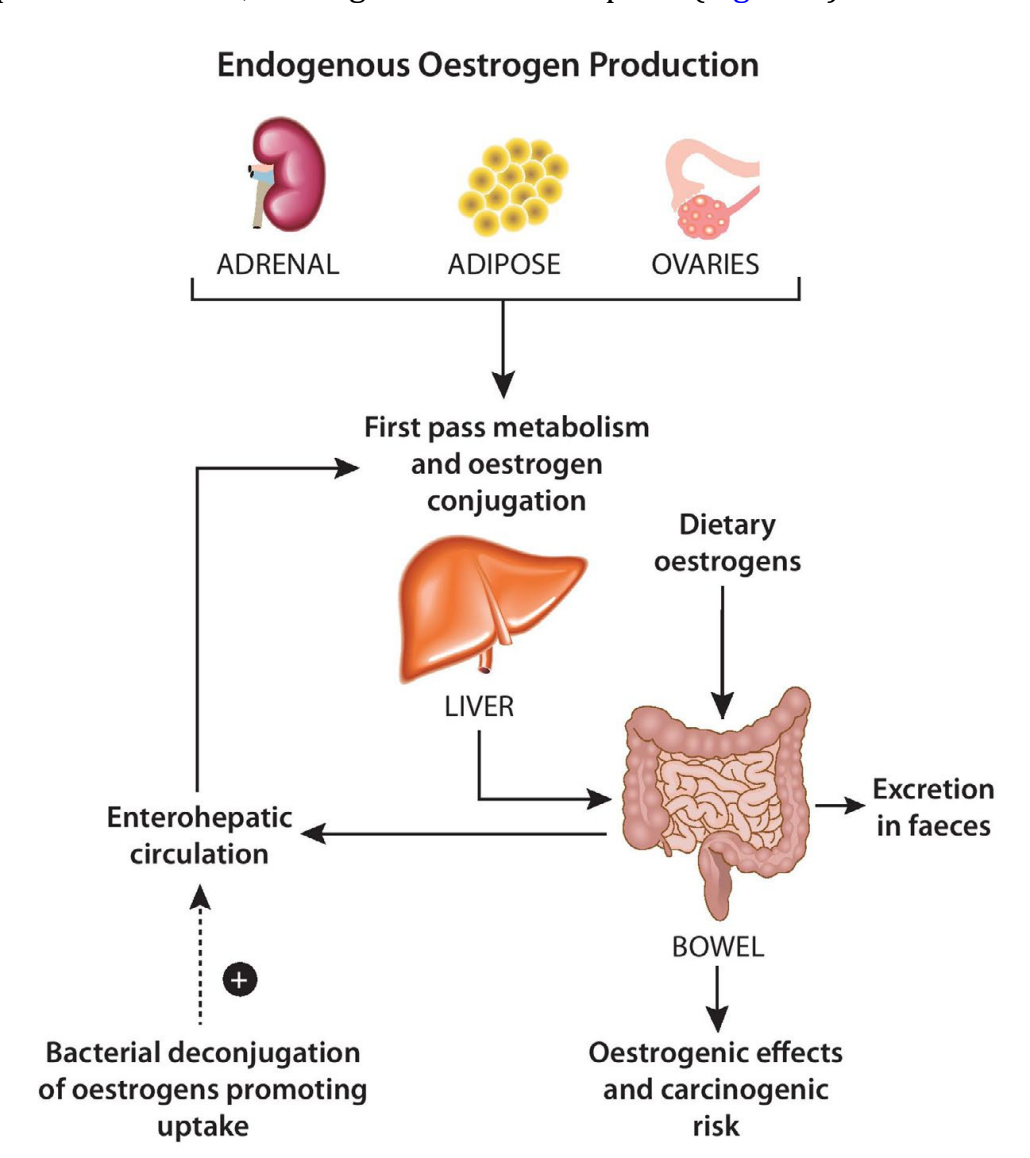
Enterohepatic circulation of oestrogens and effect of the “Estrobolome”

Endogenous oestrogen is found in a number of forms: oestradiol (the predominant form in pre-menopausal women); oestrone (predominant in post-menopausal women) an oestriol (predominant in gravid women). Collectively, these oestrogen hormones are produced predominantly within the ovaries, adrenals, adipose tissue and during their circulation they are subject to conjugation by first-pass hepatic metabolism. The conjugated oestrogens are then excreted renally or via bile into faeces. Conjugated oestrogens which are excreted into bile are subjected to the “estrobolome”. Unconjugated oestrogens within the GI tract can be reabsorbed into the circulation, and it is known that bacterial species can deconjugate oestrogens previously conjugated by first pass metabolism, leading to their reabsorption (Figure 1).

Particular bacteria have been identified to have the ability to deconjugate oestrogens via hydrolytic enzymes beta-glucuronidases and beta-glucosidases [26]. In deconjugating the oestrogens, their reuptake is promoted and therefore it is speculated that the composition of the gut microbiota can influence the risk of breast cancer (Figure 1). Women with lower plasma oestrogen concentrations have been found to have reduced faecal glucoronidase activity [27]. In the mouse model, several bacterial taxa with beta-glucoronidase activity have been shown to change upon administration of conjugated oestrogens and bazedoxifene, a common treatment for peri-menopausal symptoms [28]. In this study, Chen et al. [28] identified that faecal glucoronidase activity decreased after treatment with conjugated oestrogens and bazedoxifene. This is the first published evidence to support the ability of oestrogen therapy to modulate the gut microbiota. In relation to the treatment of breast cancer, the authors suggest that probiotic supplementation to modulate gut microbial activity may present an opportunity to alter the half-life or serum concentration of tamoxifen (TAM), a selective oestrogen receptor modulator, in order to optimise its therapeutic effect. Indeed, lower serum concentrations of the active TAM metabolite have been associated with poorer outcome in the disease [29]. However, any specific positive or negative influence of the gut microbiota on the hormonal treatment of breast cancer remains unknown.

### Immunotherapy

There is increasing evidence to suggest that the degree of clinical response to immune checkpoint inhibitors is shaped by the characteristics of the gut microbiota. The two most widely recognised immune checkpoint inhibitors are cytotoxic T lymphocyte-associated antigen 4 (CTLA-4) and programmed death-ligand 1/programmed cell death protein-1 (PD-L1/PD-1). These are emerging therapies in the treatment of breast cancer, however, evidence for the impact of the gut microbiota on these therapies exists largely in the context of metastatic melanoma [30]. Ipilimumab is a fully human monoclonal antibody directed against CTLA-4, a major negative regulator of T cell activation which improved overall survival of patients with metastatic melanoma. Anti-tumour effects of CTLA-4 blockade have been found to depend on distinct *Bacteriodes* species both in mice and human models, particularly B. *thetaiotaomicron* and B. *fragilis* being associated with efficacious CTLA-4 blockade [31].

A number of studies into anti-PD-1 and the relationship with malignant melanoma have found that treatment responders harbour more diverse gut microbiota and increased anti-tumour T cell responses compared to non-responders [32–34]. It is thought that the microbiota of immunotherapy responders may upregulate the immune response through enhancing antigen presentation, or increasing T cell recruitment in the local tumour environment. *Bifidobacterium* and *Akkermansia muciniphila* have been some of a number of specific bacterial species correlated with positive response to anti-PD-L1 immunotherapy [35].

### Other targeted therapies

Study into the impact of the gut microbiota on targeted therapies for breast cancer is in its infancy, and the gut microbiota itself has been identified as a therapeutic target. Commensal dysbiosis, the disruption of the normal gut microbiota, has been identified as a potential biomarker for high-risk breast cancer or as a potential therapeutic target in reducing tumour-promoting inflammation and subsequent metastatic dissemination [36].

Using a mouse model of hormone receptor positive mammary cancer, Buchta Rosean et al. [36] identified commensal dysbiosis as a host-intrinsic regulator of tissue inflammation, fibrosis and tumour dissemination which all are known contributors to poor survival outcomes in hormone receptor positive breast cancer. In this study, two mammary cancer cell lines were injected orthotopically into the abdominal mammary fat pad. The first used was BRPKp110 [37], a cell line for poorly metastatic hormone receptor positive mammary cancer. The second was a highly metastatic cell line, polyomavirus middle T (PyMT) [38]. Prior to this, gut dysbiosis was induced within the mouse models using an antibiotic cocktail which resulted in a significant reduction in community richness within the gut.

After tumour cell implantation, dysbiotic mice had a significant increase in total accumulated myeloid cells (including macrophages) within the normal mammary tissue adjacent to tumour. Flow cytometry was also used to evaluate for tumour cell dissemination in the poorly metastatic mouse model identifying significantly more disseminated tumour cells within axillary lymph node and lung tissue. Although it remains unknown whether host-intrinsic differences in immune function promote early dissemination of tumour cells, lymph node involvement is well known to be one of the most reliable predictors of recurrent disease and hence is often used as a marker to necessitate an upstaging of treatment [39]. Hence, future study in this field is likely to translate this work into the clinical setting with the view to using gut dysbiosis as a biomarker or therapeutic target.

### Current trials/trial evidence

A number of active clinical trials and observational studies are currently ongoing to investigate the impact of the gut microbiota on breast cancer treatments. One study, for example, is exploring the ability of gut microbiota composition to predict progression-free survival and whether there is any correlation with response to treatment [40]. Similarly, another study is investigating whether the gut microbial composition can influence immune response to the tumour, resulting in individual differences in response to anti-cancer therapies [41]. Multiple other studies are also looking at the gut microbiota composition in relation to the treatment of breast cancer. These studies are summarized in Table 1.

**Table 1.** Summary of ongoing clinical trials and observational studies in breast cancer treatment in relation to gut microbiota

**Study**	**Aims**	**Study type**	**Sample size**
“Engineering gut microbiome to target breast cancer” [42]	To determine whether probiotics will help the body’s immune system react to breast cancer	Clinical trial	7
“Gut microbiome and GI toxicities as determinants of response to neoadjuvant chemo for advanced breast cancer” [41]	To determine whether gut microbial composition can influence immune response to the tumour, resulting in inter-individual differences in the response to anti-cancer therapies	Clinical trial	40
“Intestinal microbiota of breast cancer patients undergoing chemotherapy” [43]	To study specific types (or groups) of bacteria that promote the occurrence, development, and mechanism of breast cancer	Observational	80
“Breast cancer and its relationship with the microbiota” [44]	To examine associated between composition and functionality of the mammary/gut microbiota with breast cancer risk, and to determine if exposure to environmental contaminants [endocrine disruptors, endocrine disrupting chemicals (EDCs)] can alter the microbiota?	Observational	200
“Determinants of acquired endocrine resistance in metastaticbreast cancer: a pilot study” [45]	To identify markers of endocrine resistance in ctDNA and the gut microbiome in patients with estrogen (Oestrogen) receptor positive (ER^+^) HER2^–^ metastatic breast cancer	Observational	20
“Study to investigate efficacy of a novel probiotic on the bacteriome and mycobiome of breast cancer” [46]	To examine the effect of probiotics on the breast tumour microbiome and gut microbiome in breast cancer	Clinical trial	100
“Effects of chemotherapy on intestinal bacteria in patients with newly diagnosed breast cancer” [47]	To study effects of chemotherapy on intestinal bacteria/organisms in patients newly diagnosed with breast cancer	Observational	36
“ARGONAUT: stool and blood sample bank for cancer patients” [40]	To determine whether the microbiota composition can predict progression-free survival. Whole genome sequencing and metabolomics will be used to characterize the patient’s microbiome, and whether there is any correlation with response to treatment	Observational	4,000
“Gut and intratumoural microbiome effect on the neoadjuvant chemotherapy-induced immunosurveillance in triple negative breast cancer” [48]	To determine if the probability of pathologic complete response in triple negative breast cancer patients treated with standard of care neoadjuvant chemotherapy is correlated with variability in the composition of intestinal and intra-tumoral microbiota and subsequent short-term alterations in composition	Observational	49
“The clinical study of modern therapies on flora in body fluids and blood of malignant tumour patients” [49]	To study what modern therapies lead to the influence of microecological environment including diversity and abundance of bacteria in patients who received malignant tumours	Observational	500

## Future perspective

Genetic profiling and gene-environment interactions are potential future players which can be used to target treatment in breast cancer. An interaction between CASP8-rs1045485 and alcohol consumption has been replicated in the pathogenesis of breast cancer, however, several others have not, including LSP1-rs3817198 and parity and 1p11.2-rs11249433 and ever being parous [50]. Additionally, it is not known what effect these interactions have on the gut microbiota itself.

The gut microbiota is influenced by host genetics, with the *Christensenellaceae* family of bacteria identified as the most heritable taxon [51]. This family has been shown to co-occur with other heritable bacteria and archaea. Additionally, *Christensenellaceae* and its co-inhabitants are known to be enriched in subjects with low BMI and when transplanted to germfree mice they demonstrate reduced weight gain [51]. These findings indicate a direct relationship between genetics and gut microbiota, which can potentially be targeted for breast cancer treatment.

One study has identified germline genetic polymorphisms across 24 different cancer types, which are associated with variable tissue gene expression [52]. Expression of inducible T-cell co-stimulator ligand (*ICOSLG*) and endoplasmic reticulum aminopeptidase 2 (*ERAP2*) (major regulators of immunity) were shown to be under strong genetic control and germline variants associated with a strong immune response were also defined [52]. This demonstrates that germline genetics can potentially be used to tailor immunotherapy in breast cancer patients based on the germline phenotype.

Genetic and environmental factors no doubt play a major role in the make-up of the immune system and the gut microbiota, however, specific gene-environment interactions in relation of these to breast cancer are not yet known.

As previously discussed, nutrition, lifestyle, diet, microbiome, and environment are some of the exogenous factors that influence the genome and metabolome of neoplastic and immune cells. The field of molecular pathological epidemiology (MPE) is a promising direction in investigating the role of exogenous factors in relation to the treatment of breast cancer and can provide insights into environment-tumor-host interactions. This is done by integrating pathological principles into data science fields, including bioinformatics, biostatistics, and epidemiology [53]. Novel players which aim to transform the field of MPE include artificial intelligence, digital pathology, systems biology and *in vivo* pathology techniques as we continue to advance the field of precision medicine [53]. This transdisciplinary field can contribute to improved understanding of the interactive role of immune cells, tumor cells, microbiome, and environment.

This integrative science approach can be used to investigate a variety of neoplastic and non-neoplastic diseases, and it can enhance causal inference and technological advances continuously enable more complex analyses [54]. The drawbacks include sample size limitations, requirement for interdisciplinary experts, standardized guidelines and education programs and need for rigorous validation of findings [54].

## Conclusions

A vast number of potential connections exist between the gut microbiota and malignant breast disease. However, investigation into the specific interactions is in its infancy and there is still much to understand about the effect of the gut microbiota on systemic breast cancer treatments. Our literature review highlights the role of the microbiota in influencing response to chemotherapeutic drugs and their side effects. The gut microbiota has the capacity to metabolize systemic treatments in cancer and modulate the immune system in its response to treatment. Future work will expand the evidence base with specific regard to breast cancer, and further explore the potential of gut dysbiosis to act as a biomarker and therapeutic target itself.

## Abbreviations

CTLA-4: cytotoxic T lymphocyte-associated antigen 4
GI: gastrointestinal
HER2: human epidermal growth factor receptor 2

## Declarations

### Copyright

© The Author(s) 2021.
